# Obstetric involvement in mitochondrial disorders: A review

**DOI:** 10.1097/MD.0000000000033336

**Published:** 2023-03-17

**Authors:** Josef Finsterer

**Affiliations:** a Neurology and Neurophysiology Center, Vienna, Austria.

**Keywords:** eclampsia, mitochondrial disorder, motherhood, mtDNA, oxidative phosphorylation, polyhydramnion, premature delivery, stillbirth

## Abstract

This is the first review about obstetric involvement in mitochondrial disorders (MIDs). The purpose of the review was to discuss recent advances and knowledge about the type and frequency of obstetric complications in MIDs. A narrative review for preferred reporting items was performed in MEDLINE, Current Contents, EMBASE, Web of Science, Web of Knowledge, LILACS, SCOPUS, and Google Scholar. The author searched for studies examining obstetric complications in patients with a definite MID. Obstetric complications described in MIDs include eclampsia, preeclampsia, intra uterine growth retardation, polyhydramnion, oligoamnion, decreased fetal movements, premature delivery, stillbirth, blow weakness, dystocia, breech presentation, retained placenta, postnatal hemorrhage, low birth weight, and early postnatal death. The most common of these complications are polyhydramnion, stillbirth, premature delivery, and low birth weight. The data show that some obstetric complications are more common in MIDs than in healthy females. MIDs can be associated with various obstetric complications. Some of these complications are more common in pregnant females with MID compared with healthy pregnant females. Obstetricians should be aware of MIDs and should know that pregnant females with a MID have an increased risk of developing complications during pregnancy or delivery.

## 1. Introduction

Mitochondrial disorders (MIDs) often begin as a mono-organ disease but usually become a multisystem problem as the disease progresses. Organs or tissues most commonly affected by MIDs are the central nervous system, peripheral nervous system, eyes, ears, heart, guts, kidneys, connective tissue, bone marrow, and skin. Common clinical manifestations include cognitive impairment, epilepsy, stroke-like episodes, spasticity, movement disorders (Parkinsonism, dystonia, choreoathetosis, tics, ataxia, myocloni, tremor, restless leg syndrome), cataract, optic atrophy, retinopathy, hypoacusis, cardiomyopathy, supraventricular and ventricular arrhythmias, heart failure, vomiting, diarrhea, constipation, hepatopathy, kidney stones, renal insufficiency, anemia, and psoriasis.

One of the systems often affected by MIDs is the endocrine system.^[1]^ Endocrine abnormalities in MIDs include pituitary adenoma, empty sella syndrome, short stature, hypothyroidism, hyperthyroidism, hypoparathyroidism, hyperparathyroidism, diabetes, hypoaldosteronism, hypocorticism, and hypogonadism. Hypogonadism in MIDs can have different consequences in males and females. In male’s hypogonadism can present as decreased libido, impotentia coeundi, or impotentia generandi. In female’s hypogonadism can manifest as menstrual abnormalities, polycystic ovari syndrome, decreased libido, or obstetric complications. This narrative review focuses on the obstetric complications in females with MID.

## 2. Methods

Data for this review were identified by searches of MEDLINE, Current Contents, EMBASE, Web of Science, Web of Knowledge, LILACS, SCOPUS, and Google Scholar for references of relevant articles. Terms applied for searching these databases were “mitochondrial disorder,” “mitochondrial encephalopathy, lactic acidosis, and stroke-like episodes (MELAS),” “myoclonic epilepsy with ragged-red fibers,” “chronic progressive external ophthalmoplegia,” “Kearns Sayre syndrome,” “Leigh syndrome,” or “mitochondrial DNA (mtDNA),” combined with “pregnancy,” “premature delivery,” “stillbirth,” “polyhydramnion,” “eclampsia,” “preeclampsia,” “gestational diabetes,” or “intrauterine growth retardation.” The results of the search were further screened for potentially relevant studies. Reference lists of the studies found were checked for further articles. Websites checked for additional information, particularly genetic, and to assess the pathogenicity of mtDNA mutations, were: MITOMAP https://www.mitomap.org/foswiki/bin/view/MITOMAP/ClinicalPhenotypesRNA), Neuromuscular Disease Center Database and http://neuromuscular.wustl.edu/mitosyn.html#merrf.

Only original articles about humans published between 1966 and 2023 were included. Reviews, editorials, and letters were not included. Ethical approval was waived as the data was collected solely from published literature. Altogether, 23 articles were included in the review.

## 3. Results

Obstetric abnormalities reported in MIDs include preeclampsia and eclampsia, intra uterine growth retardation, gestational diabetes, intra uterine growth retardation, placental insufficiency, polyhydramnion, premature delivery, stillbirth, blow weakness, loop of the umbilical cord, dystocia, breech position, and early postpartum death (Table 1).

**Table 1:** Obstetric complications in MIDs.

Obstetric complication	MID	Frequency	Reference
Polyhydramnion	MIMODS, FBXL4-RMID	30%	[von Kleist-Retzow et al 2003]
Stillbirth, miscarriages	MERRF, MIMODS	26%	[Kuleva et al 2018]
Premature delivery	MELAS, KSS, MIMODS	25%	[de Laat et al 2015]
Low birth weight	MIMODS	23%	[von Kleist-Retzow et al 2003]
IUGR	POLG-RMID	16%	[von Kleist-Retzow et al 2003]
Gestational diabetes	MELAS, MERRF	3.5–14%	[Khan et al 2015, Kuleva et al 2018]
Eclampsia, preeclampsia	MELAS, KSS, MIMODS	12%	[de Laat et al 2015]
Postpartal hemorrhage	MIMODS	12%	[Kuleva et al 2018]
Oligoamnion	MIMODS	10%	[von Kleist-Retzow et al 2003]
Decreased fetal movements	MIMODS	5%	[von Kleist-Retzow et al 2003]
Blow weakness	KSS	NP	[Faris et al 2014]
Dystocia	MELAS	NP	[Chou et al 2004]
Breach presentation	POLG-RMID	NP	[Inbar-Feigenberg et al 2018]
Retained placenta	MELAS	NP	[Bell et al 2017]
Early postpartum death	MIMODS	NP	[Finstreer et al 2018]

FBXL4-RMID = *FBXL4*-related mitochondrial disorder, IUGR = intra uterine growth retardation, KSS = Kearns-Sayre syndrome, MID = mitochondrial disorder, MELAS = mitochondrial encephalopathy, lactic acidosis, and stroke-like episodes, MERRF = myoclonic epilepsy with ragged-red fibers, MIMODS = mitochondrial, multi-organ disorder syndrome, NP = not provided, POLG-RMID = *POLG1*-related mitochondrial disorder.

### 3.1. Preeclampsia, eclampsia

Eclampsia is characterized by gestational hypertension, proteinuria, and edema. Preeclampsia is characterized by gestational arterial hypertension and proteinuria. In a 36-year-old gravida 2, para 0 with MELAS syndrome due to an mtDNA variant, severe preeclampsia developed during the third trimester, manifesting as severe hyponatremia, hyperkalemia, and diabetes.^[2]^ In a study of 46 females carrying the m.3243 A > G variant in *mitochondrially encoded tRNA(leucine*) (*MT-TL1*) and experiencing 98 pregnancies, 12% had preeclampsia.^[3]^ Preterm labor and pregnancy-induced hypertension was reported in a 29-year-old primigravida with Kearns-Sayre syndrome (KSS).^[4]^ The second pregnancy of this patient was uneventful.^[4]^ In a 3 generation Norwegian family with a maternally inherited mitochondrial NADH-ubiquinone oxidoreductase defect manifesting with variable hyperacusis, ataxia, weakness, fatigue, peripheral neuropathy, and retinal degeneration, reported pregnancies were associated with severe preeclampsia and eclampsia in 6 out of 10 affected females.^[5]^ Arterial hypertension has been also reported in a female with mitochondrial multi-organ disorder syndrome (MIMODS) in labor.^[6]^ Simultaneously, she was found to have proteinuria.^[6]^ Her pregnant weight was 35 kg and she delivered at term.^[6]^

### 3.2. Gestational diabetes

In a study of 46 females carrying the m.3243 A > G variant in *MT-TL1* and experiencing 98 pregnancies, 11% developed gestational diabetes during the observational period.^[3]^ In a study of 140 pregnant females with gestational diabetes the variant m.3243 A > G was detected in 11 females (8%) and the variant m.8344 A > G in 5 of 140 females (3.5%).^[7]^ In a multigravida carrying the m.3243 A > G variant, diabetes exacerbated during pregnancy requiring insulin and resulting in macrosomia of the fetus.^[8]^ Pregnancy in a female carrying the m.3243 A > G variant and manifesting with Wolff-Parkinson white syndrome, deafness, and premature graying, was complicated by gestational diabetes and placenta accreta.^[9]^ In a study of 84 Taiwanese patients with insulin-dependent diabetes, 2 carried the m.3243 A > G variant of whom 1 manifested initially as gestational diabetes.^[10]^ In a study of 57 females carrying a known mtDNA mutation or a heterozygous variant in an nDNA located gene, the frequency of gestational diabetes was 14%,^[11]^

### 3.3. Intra uterine growth retardation/restriction (IUGR)

IUGR is an obstetric complication characterized by placental insufficiency and secondary cardiovascular remodeling.^[12]^ In a retrospective review of 300 patients with proven respiratory chain defects for fetal development, based on the course of pregnancy, duration of pregnancy, antenatal ultrasonography, birth weight, birth length, and head circumference, IUGR was found in 16%.^[13]^ In a male patient carrying the *POLG1* variant c.2542 G > A, magnetic resonance imaging 2 days after birth revealed severe cerebellar atrophy, volume loss of the caudate head, putamen, and globus pallidus resulting in enlargement of the anterior horns of the lateral ventricles.^[14]^ There was also focal calcification in the thalamus bilaterally.^[14]^ The sibling of the index patient also presented with IUGR in addition to cerebellar atrophy, dysplasia of the dentate nucleus, symmetric olivary hypoplasia, hypoplasia of the dorsal columns, neonatal loss of the substantia nigra, striatum, lentiform nucleus, and ventral tier of thalamus.^[14]^ In females with a history of a pregnancy with IUGR, activity of respiratory chain complex-1 was reduced in placental tissues.^[12]^ In a study of 57 females carrying a known mtDNA mutation or a heterozygous variant in an nDNA located gene, the frequency of IUGR was 10%.^[11]^ IUGR has been also reported in a heterozygous female carrier of a *MRPS28* variant.

### 3.4. Polyhydramnion, oligoamnions

Polyhydramnion (uterine size outpacing gestational age) in the second or third trimester of pregnancy is defined as a maximum vertical pocket > 8 cm, or an amniotic fluid index > 24 cm.^[15]^ Approximately 90% of cases are idiopathic or caused by gestational diabetes.^[15]^ In a 30-year-old primigravida, polyhydramnion was diagnosed at gestational week 25 + 5 (MPV: 9,9 cm, amniotic fluid index: 27.5 cm).^[15]^ Her neonate presented with facial dysmorphism (high arched eyebrows, triangular face, upslanting palpebral fissures, and prominent chin), hypotonia, peri- and intraventricular cerebral bleeding, cerebellar atrophy, lack of subcutaneous fat, ventricular septal defect, and severe lactic acidosis.^[15]^ The patient died 2 days after birth. postmortem genetic work-up by exome sequencing revealed the nonsense mutations c.292C > T (p.[Arg98*]) and c.1303 C > T (p.[Arg435*]) in *FBXL4* (Table 2).^[15]^ The corresponding gene encodes a member of the F-box protein family, which is characterized by an approximately 40 amino acid motif, the F-box. The F-box domain mediates protein-protein interactions and binds directly to S-phase kinase-associated protein-1. Mutations in *FBXL4* cause mitochondrial myopathy.^[15]^ In a retrospective review of 300 patients with proven respiratory chain defects, polyhydramnion was found in 30% of the cases and oligoamnions in 10% of the cases.^[13]^

**Table 2:** Mutations in mtDNA or nDNA located genes which have been associated with obstetric complications.

Mutation	Gene	Obstetric involvement
m.3243A > G	MT-TL1	Eclampsia, preeclampsia, gestational diabetes, premature delivery
m.8344A > G	MT-TK	Stillbirth, gestational diabetes
m.1555A > G	MT-RNR1	Preterm delivery
c.2542G > A	POLG1	Breech presentation, preterm delivery, intra uterine growth retardation
c.292C > T	FBXL4	Polyhydramnion
c.1303C > T	FBXL4	Polyhydramnion
m.4917A > G	MT-ND2	Premature delivery
m.4216T > G	UC	Premature delivery
m.10398G > A	MT-ND3	Premature delivery
mtDNAdel	Multiple	Preeclampsia, preterm birth, blow weakness
mtDNA depletion	Multiple	Decreased fetal movements

mtDNA = mitochondrial DNA, *MT-TL1 = mitochondrially encoded tRNA(leucine*), UC = uncharacterized.

### 3.5. Decreased fetal movements

In a retrospective review of 300 patients with proven respiratory chain defects for fetal development, decreased fetal movements were found in 5%.^[13]^ Two sisters developed diminished fetal movements during the third trimester in addition to skin edema.^[16]^ Shortly after birth both developed lactic acidosis with multi-organ failure.^[16]^ Muscle biopsy in these patients revealed markedly reduced activity of various respiratory chain complexes. The intramitochondrial amount of mtDNA was markedly decreased in these patients, which is why an mtDNA depletion syndrome was diagnosed.^[16]^

### 3.6. Premature delivery (spontaneous preterm birth)

Spontaneous preterm birth is defined as spontaneous delivery before gestational week 38.^[17]^ In a study of 46 females carrying the m.3243 A > G variant in *MT-TL1* and experiencing 98 pregnancies, 25.3% had a premature delivery and 5 of them had a gestation period < 32 weeks.^[3]^ In a male carrying the *POLG1* variant c.2542 G > A premature delivery at gestational week 35 + 3 was associated with seizures immediately after delivery and dysmorphism (microcephaly, sloped forehead, hypotelorism, and micrognathia).^[14]^ In a prematurely born male with lactic acidosis and hypertrophic cardiomyopathy, the variant m.3243 A > G was identified as causative.^[18]^ Preterm labor was reported in a 29-year-old primigravida with KSS.^[4]^ In a study of 7056 infants with a history of preterm delivery, the variant m.1555 A > G was detected in 12 of them (Table 2).^[19]^ In a sample of 220 pregnant females with a history of preterm delivery, an association between preterm birth and the mtDNA variants m.4917A > G, m.4216T > G, and m.10398 G > A was established.^[17]^ According to these data premature delivery is an occasional complication of pregnancies in females with a MID. Whether the rate of preterm delivery in MID patients is truly elevated compared to healthy or diseased controls requires further investigations.

### 3.7. Stillbirth, spontaneous abortion, miscarriage

In an Indian family with myoclonic epilepsy with ragged-red fibers syndrome due to the variant m.8344 A > G in *MT-TK*, the mother of the index case had a history of triple abortion always after 3 months of pregnancy and neonatal death of the biovular twin of the index case.^[20]^ The mother of a 27-year-old female with MIMODS, who was suspected to have transmitted the MID to her offspring, had an individual history of recurrent stillbirths.^[21]^ In a 3 generation Norwegian family with a maternally inherited mitochondrial NADH-ubiquinone oxidoreductase defect, the incidence of spontaneous abortion, preterm births, and stillbirths was significantly increased.^[5]^ In a study of 57 females carrying a known mtDNA mutation or a heterozygous variant in an nDNA located gene, the frequency of terminations of pregnancy was 20%, the percentage of life births 52%, and the frequency of miscarriages 26%.^[11]^ According to these data, abortions and stillbirths are frequent complications of pregnancies in females with a MID. It remains questionable whether stillbirths in these patients are due to MID in the fetus or due to MID in the pregnant female.

### 3.8. Blow weakness

In a 26-year-old primigravida with KSS the baby had to be delivered by Cesarean section in gestational week 36 because of failure of labor progression.^[22]^

### 3.9. Dystocia

In a prenatally diagnosed fetus with MELAS syndrome, the mother, who also suffered from MELAS, developed gestational diabetes, and the fetus macrosomia.^[8]^ Delivery of the fetus was complicated by shoulder dystocia.^[8]^

### 3.10. Breach presentation

In a male carrying the *POLG1* variant c.2542 G > A, Cesarean section had to be carried out because of breech presentation.^[14]^

### 3.11. Retained placenta

In a 36-year-old pregnant female with MELAS syndrome a retained placenta developed postpartum with postpartum hemorrhage requiring urgent instrumental delivery under spinal anesthesia, transfusion, and admission to the intensive care unit for pulmonary edema, effusions, and atelectasis.^[2]^ In a study of 57 females carrying a known mtDNA mutation or a heterozygous variant in an nDNA located gene, the frequency of postpartum hemorrhage was 12%.^[11]^

### 3.12. Low birth weight

In a retrospective review of 300 patients with proven respiratory chain defects for fetal development, based on course of pregnancy, duration of pregnancy, antenatal ultrasonography, birth weight, birth length, and head circumference, low birth weight was found in 23% of the cases.^[13]^

### 3.13. Early postpartum death

In a report on a 27-year-old female with MIMODS manifesting with myopathy, polyarthralgia, cutaneous manifestations, osteoporosis, pancreatitis, anemia, migraine-like headache, nephrolithiasis, ovarian cysts, depression, right bundle branch block, AV-block III, sialadenitis, hyperlipidemia, nephrolithiasis, patella luxation, and hypogonadism, the family history was positive for death of her only sibling 5 days after delivery.^[21]^

### 3.14. Others

In addition to the obstetric complications discussed above, the frequency of terminations of pregnancy is higher and the percentage of life birth is reduced compared to controls in females carrying a pathogenic mtDNA variant.^[11]^ In a 29-year-old Indian primigravida, MELAS manifested for the first time as focal epileptic state in gestational week 10.^[23]^ Seizures were sufficiently controlled with carbamazepine.^[23]^ A 31-year-old primigravida developed myopathy and neuropathy during pregnancy and was subsequently diagnosed with MELAS due to the variant m.3271 T > C.^[24]^ Despite the onset of MELAS during pregnancy she delivered a healthy boy.^[24]^

## 4. Discussion

This review shows that MIDs can be associated with a variety of obstetric complications. Some of the complications are increased in pregnant females with MID compared to healthy pregnant females. Obstetric complications previously described in MIDs include eclampsia, preeclampsia, intra uterine growth retardation, polyhydramnion, oligoammnion, decreased fetal movements, premature delivery, stillbirth, blow weakness, dystocia, breech presentation, retained placenta, postnatal hemorrhage, low birth weight, and early postnatal death. The most common of these complications are polyhydramnion, stillbirth, prematurity, and low birth weight. However, data on the frequency of obstetric complications in MID patients are limited to a few studies. Therefore, further prospective studies should be conducted to assess the true incidence and nature of obstetric complications in MIDs.

To explain the apparently increased frequency of obstetric complications in females with MIDs it can be speculated that energy deprivation, oxidative stress, or increased apoptosis could play a significant pathophysiological role. Nonetheless, these speculations need to be verified in basic and clinical studies on the relationship between pregnancy and the underlying mitochondrial metabolic defect.

Since preterm labor is not uncommon, particularly in smaller females,^[25]^ and arterial hypertension is common, particularly in primipara,^[26]^ a causal relationship between MIDs and preterm labor or eclampsia cannot be readily established. However, because arterial hypertension can be a primary manifestation of MIDs,^[27]^ and because females with MID appear to be prone to obstetric complications, it is highly likely that arterial hypertension during pregnancy or labor can be a primary manifestation of the underlying mitochondrial defect.

Because females with MID can survive into adulthood, and some MIDs begin in adulthood, examining the relationship between MIDs and obstetric complications is critical. We need to know more about these relationships, why as a first step, pregnancies of MID females should be closely monitored and any complications during pregnancy should be reported. In a second step, pregnancies of MID females should be prospectively examined for the type, frequency, and cause of possible obstetric complications.

## Author contribution

**Conceptualization:** Josef Finsterer.

**Data curation:** Josef Finsterer.

**Resources:** Josef Finsterer.

**Writing – original draft:** Josef Finsterer.
